# Prevalence, Timing and Mutual Relationships of Acute and Chronic Respiratory Failure in People with Chronic Obstructive Pulmonary Disease

**DOI:** 10.3390/jcm15145595

**Published:** 2026-07-16

**Authors:** Michele Vitacca, Gaia Riboni, Mara Paneroni, Valentina Tibollo, Riccardo Bellazzi, Nicolino Ambrosino

**Affiliations:** 1 Respiratory Rehabilitation Unit of the Institute of Lumezzane, Istituti Clinici Scientifici Maugeri IRCCS, 25065 Lumezzane, Italy; mara.paneroni@icsmaugeri.it; 2 Laboratory of Medical Informatics and Artificial Intelligence of the Institute of Pavia, Istituti Clinici Scientifici Maugeri IRCCS, 27100 Pavia, Italy; gaia.riboni@icsmaugeri.it (G.R.); valentina.tibollo@icsmaugeri.it (V.T.); riccardo.bellazzi@icsmaugeri.it (R.B.); 3 Department of Electrical, Computer and Biomedical Engineering, University of Pavia, 27100 Pavia, Italy; 4 Respiratory Rehabilitation Unit of the Institute of Montescano, Istituti Clinici Scientifici Maugeri IRCCS, 27040 Montescano, 27100 Pavia, Italy; nico.ambrosino@gmail.com

**Keywords:** COPD exacerbation, COPD acute respiratory failure, chronic respiratory failure

## Abstract

**Background and aim:** Prevalence, time to first diagnosis of acute (ARF) and chronic (CRF) respiratory failure in people with chronic obstructive pulmonary disease (COPD) are still unclear and were assessed in this 15-year, retrospective, observational, real-world data study. **Methods:** Our study examined one cohort (Cohort C1) of people older than 40 years diagnosed with COPD without any previous diagnosis of ARF. To assess the temporal relationship between ARF and CRF, two more cohorts of individuals were created: Cohort C2 included people with COPD subsequently diagnosed with CRF, without any previous diagnosis of ARF. Cohort C3 consisted of COPD people subsequently diagnosed with ARF, without any previous diagnosis of CRF. Adjusted hazard risk of and timing to first diagnosis were estimated for: i-ARF and CRF in Cohort C1; ii-ARF in Cohort C2; iii-CRF in Cohort C3. **Results:** Participants in Cohort C1 were equally distributed by gender, with a mean age of 66.9 ± 12.2 years and BMI 28.1 ± 7.72 kg/m^2^. After 15 years since COPD diagnosis, participants in Cohort C1 showed 45.25% (75th percentile reached after 7.2 years) and 21.86% (80th percentile after 13.7 years) probabilities for ARF and CRF, respectively. Participants in Cohort C2 showed an 86.78% ARF (acute-on-chronic) probability (1.7 years median time to ARF since CRF diagnosis). Individuals in Cohort C3 showed a 41.52% CRF probability (75th percentile after 3.3 years since first ARF diagnosis). **Conclusions:** COPD individuals reported different prevalence of and times to ARF or CRF diagnoses. Time since CRF diagnosis to first acute-on-chronic respiratory failure is shorter than time to post-ARF chronic respiratory failure. Our findings provide useful insights into periods of increased vulnerability following COPD diagnosis and respiratory failure events, which may have implications for patient monitoring and preventive strategies.

## 1. Introduction

Chronic obstructive pulmonary disease (COPD) is a main cause of morbidity and mortality worldwide with a 10.6% world prevalence expected to rise to 23% by 2050 [1]. With disease progression people’s lung function and health-related quality of life worsen with progressive worsening of dyspnoea, cough, and reduced exercise capacity [2]. Affected individuals may suffer also of COPD exacerbations (ECOPD) potentially resulting in acute respiratory failure (ARF).

A late event in the natural history of COPD is chronic respiratory failure (CRF), as defined by chronic hypoxaemia (Type 1) with or without hypercapnia (Type 2) [3]. In COPD, chronic hypoxaemia (PaO_2_ < 60 mmHg) contributes to mortality and has been associated with complications such as systemic inflammation and pulmonary hypertension. The need for long-term oxygen therapy (LTOT) is one of the most powerful determinants of mortality in COPD. Up to 25% of people with COPD at GOLD stages 3 and 4 may also suffer from chronic hypercapnia (PaCO_2_ > 45 mmHg). Additional treatment of Type 2 CRF may include long-term non-invasive ventilation (NIV). Both LTOT and NIV can improve survival in selected COPD populations [3].

The prevalence of CRF is reported to be relatively low in the general COPD population, increasing significantly with worsening of airflow limitation and clinical severity. However, the time from initial COPD diagnosis to the onset of CRF is highly variable and depends on the rate of disease progression, comorbidities, ECOPD frequency, and individual factors, including continued exposure to risk factors like tobacco smoke and/or environmental pollutants [2,4,5].

The prevalence of and the time to CRF onset following an ECOPD with ARF are not well defined in the current medical literature. Long-term oxygen therapy (LTOT) at discharge, a marker of CRF, is required in a notable proportion of individuals after hospitalisation due to ECOPD [6]. While exact prevalence rates vary by cohorts and definitions, studies suggest that CRF develops in a substantial minority of individuals—potentially 20–40%—after an episode of ARF requiring hospitalisation, especially in those with severe underlying disease and comorbidities [6,7].

Data from large cohorts indicate that severe ECOPD requiring hospital admission—often accompanied by acute-on-chronic respiratory failure—occur in approximately 10–20% of COPD individuals each year, with even higher rates among those with advanced disease. However, no established time interval between the onset of CRF and the following ARF onset is reported [8,9]. The mutual relationships among COPD, the onset time of ARF, CRF, and subsequent episodes of ARF have implications on both clinical and functional deterioration of affected people as well as on organisational needs and modalities of care. Anticipating the timing and magnitude of these healthcare challenges can strongly inform and motivate effective prevention and treatment strategies.

Real-world data (RWD) is becoming increasingly important for healthcare decisions when randomised controlled trial (RCT) data are lacking. It can potentially work as an alternative to cohort studies of the potential risks or lifestyle factors that cannot be randomised [10]. Our large, long-term RWD study investigates prevalence, time to diagnosis, and mutual time relationship of ARF and CRF in COPD individuals.

## 2. Materials and Methods

### 2.1. Participants

This study used RWD from the TriNetX Analytics Network (TriNetX LLC, Cambridge, MA, USA), a Global Collaborative Network, the methodology and application of which have been recently described [11]. Data were extracted from the network which contains information on over 100 million individuals, 4 million of whom coded as having COPD (see below). We assessed timing of ARF and CRF over a 15-year period starting one day after the index event, comparing the risks of ARF or CRF following a COPD diagnosis and their mutual time relationships. Data from some of these participants has been previously published elsewhere [12]. As the network data are aggregated and anonymized, Ethical Committee approval and informed consent were waived; however, the study was approved by the Local Review Board (CTSM-RC-1/2025, November 2025).

### 2.2. TriNetX

In brief, TriNetX is a network that integrates de-identified electronic medical records from over 150 healthcare organisations (HCOs) worldwide [13]. Available data include demographics, coded diagnoses (International Classification of Diseases (ICD), version 10), procedures (ICD-10 Procedure Coding System or Current Procedural Terminology), medications (National Library of Medicine and the Department of Veterans Affairs), and measurements (laboratory tests and body mass index (BMI), Logical Observation Identifiers Names and Codes). No lung functional data or causes of hospital admission are available. Further details, as well as the basis of data analysis, are shown in the Supplementary Materials.

### 2.3. Cohorts

Our study examined one cohort (Cohort C1) of people older than 40 years diagnosed with COPD (ICD-10-CM code J44) without any previous diagnosis of ARF (ICD-10-CM codes J96.0, J96.9, J96.2) or CRF (ICD-10-CM codes J96.1). The index day was the date of the first coded COPD diagnosis. To assess the temporal relationship between ARF and CRF, two more cohorts of individuals were created: Cohort C2 included people with COPD subsequently diagnosed with CRF, without any previous diagnosis of ARF. The index day was the date of the first diagnosis of CRF. Cohort C3 consisted of COPD people subsequently diagnosed with ARF, without any previous diagnosis of CRF. The index day was the date of the first ARF diagnosis. More details are provided in the Supplementary Materials.

### 2.4. Covariates

Data of age, sex, coded comorbidities, respiratory support, ventilation procedures, oxygen therapy, BMI and medications: inhaler anti-inflammatories (RE101), inhaler sympathomimetic bronchodilators (RE102), oral sympathomimetic (RE103), oral xanthine (RE104) and inhaler anticholinergic (RE105) were searched and recorded. More details are provided in the Supplementary Materials.

### 2.5. Outcomes

We analysed the risk of developing ARF and CRF.

### 2.6. Statistical Analysis

All analyses were generated with TriNetX platform software on 4 November 2025. Numerical characteristics are shown as mean and standard deviations (SDs) while categorical characteristics are shown as numbers and percentages (%) of the cohort. Baseline characteristics refer to a representative subsample (#935,483 participants) of the starting cohort. In order to improve performance times, large cohort results are limited by TriNetX Platform to approximately 10,000 participants per HCO. These are not true random samples, but arbitrary subsets used to optimise tabulation speed. The selection of a subsample is a standard procedure within the TriNetX platform and does not affect the validity of the results generated by the analysis [11]. This subsample has been used only for Table 1, while the main time-to-event analyses were performed on the full cohorts. To improve performance times for running and returning outcomes analytics results, the analysis excluded people who had met the index event criteria more than 20 years ago. This is done knowing that the vast majority of data available through TriNetX comes from patient encounters that occurred within the last 20 years. See Supplementary Materials for details.

The ‘Analyse Outcome’ function (see Supplementary Materials) was used to assess the risk and time to diagnosis. In particular, three separate outcome analyses were performed. The first, performed on the main Cohort C1, analysed the risk of developing ARF or CRF following the first COPD diagnosis. In addition, to assess the reciprocal relationship between ARF and CRF, the following analyses were performed:

- In **Cohort C2** we analysed the risk of developing ARF following a CRF diagnosis in COPD;
- In **Cohort C3**, we analysed the risk of developing CRF following an ARF diagnosis in COPD.

Risk analysis and Kaplan–Meier (KM) curves were performed. The median follow-up time, interquartile range (IQR) and time to reach specific quartiles were calculated. Lost to follow-up participants were censored on the day after the last fact in their record. Death was not treated as a censoring event. For every analysis, a 15-year time window starting 1 day after the index event was selected. Furthermore, the pre-index event window was narrowed to 3 years to better capture conditions potentially related to the index event while excluding distant, unrelated factors. To minimise potential bias and confounding factors, several sensitivity analyses were conducted [14]. Specifically, we computed the risk of ARF and CRF in the COPD cohort, excluding outcome events occurring immediately after the index event to reduce the likelihood of including pre-existing conditions. Analyses were repeated on day one and 6 months after the index event to evaluate the temporal effect. Details are provided in the Supplementary Materials.

## 3. Results

Cohort **C1** (COPD alone) consisted of 3,187,946 participants matching the query criteria from 157 HCOs. These participants had an index event that occurred within the last 20 years. They showed a median 2.9-year follow-up (IQR: 5.7 years). Participants in Cohort C1 were equally distributed by gender, with an age of 66.9 ± 12.2 years and BMI 28.1 ± 7.72 kg/m^2^.

The clinical characteristics of a representative subsample of 935,483 participants (see the Section 2.6.) are shown in Table 1 (see also Supplementary Materials for details). Interestingly, as shown in Table 1, the data on oxygen administration, a marker of ARF or CRF, appeared to be significantly lower than expected. This is likely because oxygen is classified as a “medication” rather than a “procedure”, so it might have been neither recorded as a respiratory support nor as a medication.

**Table 1:** Clinical characteristics of a representative sample (N = 935,483) of the population in the study at first COPD diagnosis.

Variables	Population
**Comorbidities, n (%)**	
Nicotine dependence	190,600 (20.37)
Essential (primary) hypertension	427,594 (45.70)
Chronic kidney disease	10,910 (1.16)
Ischemic heart diseases	255,216 (27.28)
Acute myocardial infarction	56,837 (6.07)
Atrial fibrillation and flutter	149,715 (16.00)
Unspecified atrial flutter	20,784 (2.22)
Acute-on-chronic diastolic (congestive) heart failure	27,337 (2.92)
Neoplasms	280,152 (29.95)
**Prescribed Medications, n (%)**	
Anti-inflammatories, inhalation	315,854 (33.76)
Bronchodilators, sympathomimetic, inhalation	449,917 (48.09)
Bronchodilators, sympathomimetic, oral	400,426 (42.80)
Bronchodilators, xanthine derivative	44,441 (4.75)
Bronchodilators, anticholinergic	351,601 (37.58)
Oxygen therapy	2895 (0.31)
**Procedures during Hospital stay, n (%)**	
CPAP	53,066 (5.67)
NIV	1630 (0.17)
EI + MV	43,233 (4.62)

**Legend**: Data shown as n (%). **Abbreviations**: CPAP, continuous positive airway pressure; EI, endotracheal intubation; MV, mechanical ventilation; NIV, non-invasive ventilation.

**Figure 1:**
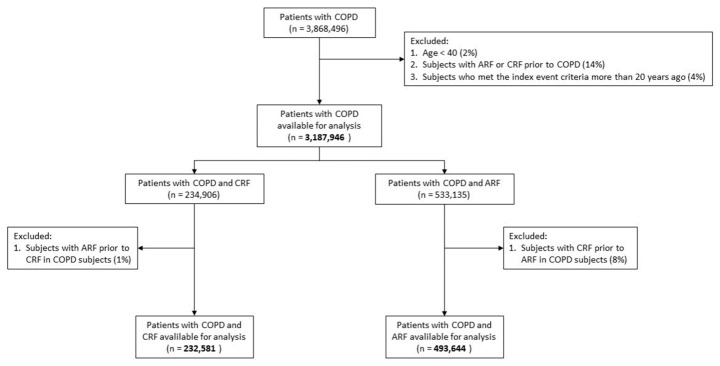
Trial profile.

Cohort **C2** (COPD with CRF followed by an ARF episode: acute-on-chronic respiratory failure) consisted of 232,581 participants from 139 HCOs, (median follow-up: 1.6 years, IQR: 3.1 years). Cohort C3 (COPD with an ARF event followed by CRF) consisted of 493,644 participants from 147 HCOs, (median follow-up: 1.2 years, IQR: 3.2 years). These two cohorts both experienced an index event within the last 20 years. Details about the sizes of the cohorts are shown in Figure 1.

**Figure 2:**
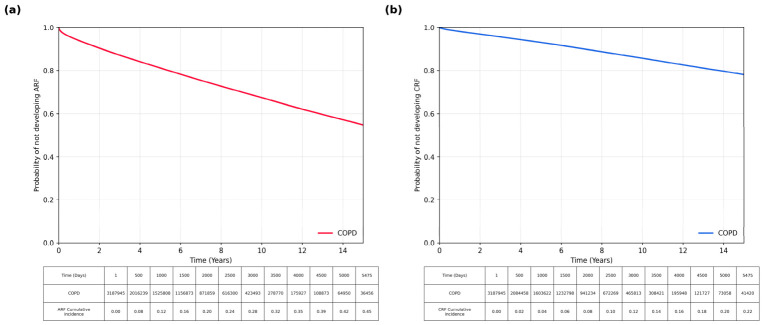
The 15-year event-free Kaplan–Meier curve and cumulative incidence for ARF (panel (**a**)) and 15-year event-free Kaplan–Meier curve and cumulative incidence for CRF in COPD individuals (panel (**b**)). **Legend**: **COPD**, chronic obstructive pulmonary disease; **ARF**, acute respiratory failure.After 15 years from COPD diagnosis, there was a 45.25% probability of developing ARF; the 75th percentile was reached after 7.2 years (panel (**a**)). There was also a 21.86% probability of developing CRF. Since the 75th percentile was not reached within the time window, the 80th percentile was calculated instead. This was reached after 13.7 years (panel (**b**)).

Figure 2a shows the 15-year event KM curve for ARF in Cohort C1. After 15 years from the first coded COPD diagnosis, there was a 45.25% probability of developing ARF, the 75th percentile reached after 7.2 years. Figure 2b shows the 15-year event-free KM curve for CRF in Cohort C1. After 15 years since the first COPD diagnosis, there was a 21.86% probability of developing CRF. Since the 75th percentile was not reached within the time window, the 80th percentile was calculated instead. This was reached after 13.7 years.

**Figure 3:**
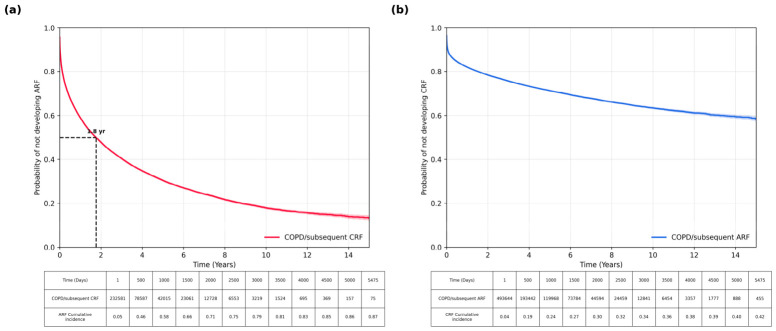
The 15-year event-free Kaplan–Meier curve and cumulative incidence for ARF (panel (**a**)) and 15-year event-free Kaplan–Meier curve and cumulative incidence for CRF in COPD + ARF individuals (panel (**b**)). **Legend**: **COPD**, chronic obstructive pulmonary disease; **ARF**, acute respiratory failure; **CRF**, chronic respiratory failure. KM analysis in people with COPD and CRF. There was an 86.78% probability of developing ARF (acute-on-chronic) within the observation period (Cohort C2). A median of 644 days (1.7 years) elapsed between the coded CRF diagnosis and the first coded ARF diagnosis (panel (**a**)). In COPD individuals with a subsequent diagnosis of ARF (Cohort C3), there was a 41.52% probability of developing CRF within the observation period. The 75th percentile was reached after 3.3 years from the ARF diagnosis (panel (**b**)).

As shown in Figure 3a, the KM analysis in people with COPD and CRF showed an 86.78% probability of developing ARF (acute-on-chronic) within the observation period (Cohort **C2**). A median of 644 days (1.7 years) elapsed between the coded CRF diagnosis and the first coded ARF diagnosis.

Finally, in COPD individuals with a subsequent diagnosis of ARF (Cohort **C3**), there was a 41.52% probability of developing CRF within the observation period. As shown in Figure 3b, the 75th percentile was reached after 3.3 years from the ARF diagnosis.

Sensitivity analyses showed fewer overall events and lower outcome probabilities across all cohorts (see Table S1 in Supplementary Materials). In the overall COPD cohort, the risk of ARF was highest shortly after diagnosis, with the probability of ARF decreasing to 42.89% after exclusion of early events. A similar but less pronounced trend was observed for CRF, with the probability of CRF decreased to 20.91%. Among participants with COPD who subsequently developed CRF, the probability of ARF decreased to 76.71% after shifting the observation window to 6 months. Likewise, among participants with COPD and prior ARF, the probability of CRF decreased to 26.41% after exclusion of early events.

These results indicate that a substantial proportion of ARF episodes and CRF occur in the first few months after the index event, suggesting the existence of a period of particular clinical vulnerability in the early stages of the natural history of the disease (see Supplementary Materials for details).

## 4. Discussion

This large, long-term, RWD study found that within the observation period in individuals with COPD, diagnoses of ARF and CRF show different prevalences and times to their first code. The findings provide useful insights into periods of increased vulnerability following COPD diagnosis and respiratory failure events, which may have clinical and organisational implications on individual monitoring and health systems’ preventive strategies.

### 4.1. TriNetX

The TriNetX database used for this study enabled large-scale analysis of the prevalence and incidence of events in large populations [10,12]. TriNetX data has been used in studies on long-term cardiovascular outcomes in COVID-19 survivors, autoimmune disorders or cardiovascular comorbidities in individuals with COPD [11,15,16,17].

### 4.2. Chronic Respiratory Failure

The prevalence of CRF within the observation period was about 22% with a median time to diagnosis longer than 15 years, confirming on a real-life basis previous observation. In the literature, in COPD individuals, the time frame for developing CRF is reported as highly variable, but longitudinal cohort data suggest that CRF most commonly develops over a period of approximately 5 to 10 years following the COPD diagnosis, particularly in people with higher baseline impairment and accelerated decline in gas exchange [18]. Most individuals developing CRF are reported to have advanced disease, often with FEV_1_ < 50% predicted [19,20]. The progression to CRF is accelerated in people with frequent ECOPD, severe airflow limitation, and comorbidities such as heart failure or pulmonary vascular disease [5]. Although the lack of physiological data such as FEV_1_ in our RWD study prevents any comparison, our results confirm CRF as a late event in people with COPD.

### 4.3. Acute Respiratory Failure

Our study also shows that the median time to the first coded ARF diagnosis exceeded 15 years after the initial COPD diagnosis, with resulting prevalence below 50%. In the medical literature, the time to the first ARF diagnosis following the diagnosis of COPD is highly variable and not defined by a specific interval. The risk is influenced by disease severity, comorbidities, and individual factors. Epidemiologic data indicate that among people with COPD, the incidence rate of ARF is approximately 3.5 per 100 person-years, with a mean follow-up of 3.5 years, but this does not translate into a predictable time from diagnosis to the first episode for the individual case [21]. Therefore, our real-life study contributes to knowledge in this field.

### 4.4. Chronic After Acute Respiratory Failure

The risk analysis showed that 41.52% of participants developed CRF after an ARF diagnosis within the observation period. The time frame for the development of CRF after an episode of ARF in people with COPD is also reported as highly variable, but CRF may become clinically apparent within weeks to months following an acute event, particularly in those with incomplete recovery of lung function or persistent gas exchange abnormalities. However, in evaluating our results, we must be aware that derangements in arterial blood gases after an episode of ARF (e.g., hypercapnia after an ECOPD) may be transient. Indeed, GOLD recommends reassessing the need for LTOT and re-evaluating gas exchange 1–4 weeks after hospital discharge for ARF, and again at 12–16 weeks. This timeframe reflects the period during which CRF may emerge or be confirmed [2]. This approach is based on the observation that some individuals do not return to their pre-exacerbation baseline and may have persistent hypoxaemia or hypercapnia, necessitating LTOT and/or ventilatory support [22].

### 4.5. Acute-on-Chronic Respiratory Failure

The risk analysis showed that 86.84% of COPD participants developed ARF following CRF diagnosis (acute-on-chronic) within the observation period. The progression to acute-on-chronic respiratory failure depends on multiple factors, including baseline severity of airflow limitation, comorbidities, frequency and severity of ECOPD, the rate of decline in gas exchange, and last but not least the management of ECOPD. There is no consensus guideline from any professional society specifying a typical time span from COPD diagnosis to acute-on-chronic respiratory failure; therefore, our study adds useful information in this field [18,23].

### 4.6. Limitations

Our results should be considered with caution. The analyses demonstrate temporal sequences but cannot address causal questions of clinical importance. The highest prevalence of CRF is reported in people with GOLD stage IV or GOLD groups C or D, meaning that advanced disease is the primary risk factor for CRF in COPD [2,4]. Unfortunately, due to the nature of the data only reporting the coded diagnoses, we were unable to match the studied populations for any level of airflow limitation or gas exchange or distinguish between the CRF types. Furthermore, information on specific management of either ARF with oxygen or NIV or of CRF with LTOT and/or NIV was available only in a few cases.

Another significant and potentially more important limitation of this study is the analysis of COPD individuals as a homogeneous entity. Due to the lack of physiological or clinical information, we were compelled to analyse participants across all severities, comorbidities, and clinical trajectories. This resulted in considering timing and mutual relationships of ARF and CRF as uniform across the large, heterogeneous COPD population. However, people with COPD likely exhibit distinct subphenotypes (e.g., frequent exacerbators, emphysema-predominant, chronic bronchitic) that may have fundamentally different risks and temporal patterns of developing respiratory failure [24,25]. Our study suffers from the limitations of a retrospective study. For instance the TriNetX Analytics Network does not collect data on events occurred at home. However, it represents real-world conditions, with a vast sample and long observation. Despite RCTs are still the cornerstone for clinical research, they have limitations too, such as strict eligibility criteria, resource request, and focus on short term outcomes, thus decreasing their applicability in real-world settings [26].

Results may suffer from confounding by unknown/unreported data such as ECOPD history, possible use of home NIV and/or LTOT in individuals with CRF. Furthermore, differences in coding habits by providers (like missing reports on oxygen therapy), nutritional and smoking habit, as well as potential differences in availability of diagnostic and therapeutic facilities, may have influenced coded diagnoses [27]. To minimise these potential bias and confounding factors, several sensitivity analyses were conducted. The results indicated that a substantial proportion of ARF episodes and CRF occur in the first few months after COPD diagnosis or immediately after a new ARF or CRF diagnosis, suggesting the existence of particular clinical vulnerability and frailty immediately after a new disease natural history step.

## 5. Conclusions

In conclusion, with the above limitations, this study has found that in individuals with a COPD diagnosis, ARF and CRF show different prevalence and time to code. These results indicate the need for frequent monitoring of arterial blood gases along the whole natural history of disease. Future work must leverage subphenotype approaches to identify individuals at the highest risk for rapid deterioration in order to inform targeted monitoring strategies.

## Data Availability

The network data used in this study are aggregated and anonymised; therefore, they are not available for any purpose.

## Supplementary Materials

The following supporting information can be downloaded at: https://www.mdpi.com/article/10.3390/jcm15145595/s1, Table S1: Sensitivity Analysis.

## Abbreviations

The following abbreviations are used in this manuscript:

ARF	Acute respiratory failure
CRF	Chronic respiratory failure
ECOPD	COPD exacerbations
COPD	Chronic obstructive pulmonary disease
LTOT	Long-term oxygen therapy
RWD	Real-world data
RCT	Randomised controlled trial
TriNetX	TriNetX Analytics Network
HCOs	Healthcare organisations
BMI	Body mass index
ICD	International Classification of Diseases
ICD-10 procedures	ICD-10-Procedure Coding System
KM	Kaplan–Meier
IQR	Interquartile range
