# Supplementary material for: Prevalence, Timing and Mutual Relationships of Acute and Chronic Respiratory Failure in People with Chronic Obstructive Pulmonary Disease
Source: J Clin Med. 2026 Jul 16;15(14):5595. doi: 10.3390/jcm15145595 (PMC13412521; doi:10.3390/jcm15145595)
Supplement: Supplementary file 1 [file jcm-15-05595-s001.zip › jcm-4388739-supplementary.pdf]

Prevalence, timing and mutual relationships of acute and chronic respiratory failure in people with chronic obstructive pulmonary disease

Supplementary Materials

TriNetX

In brief, TriNetX (TriNetX LLC, Cambridge, MA, USA) is an extensive, growing global network that integrates de-identified electronic medical records from over 150 healthcare organisations (HCOs) in different countries worldwide. TriNetX is certified to the ISO 27001:2022 standard.

The platform is a federated network which facilitates collaboration between industry, including pharmaceutical companies and research organisations and academic and community-based HCOs. TriNetX has a security and governance model that provides a federated platform of real-world data (electronic health records, datasets).

Healthcare organisations interested in TriNetX can submit an application, which undergoes an evaluation including the assessment of data quality, technical infrastructure, and data governance policies. If the required standards are satisfied, a membership agreement is established, taking into account regulations such as the European Union (EU) General Data Protection Regulation (GDPR) or the Health Insurance Portability and Accountability Act (HIPAA) in the United States (US). Each HCO is responsible for maintaining and routinely updating its data into the TriNetX model. Analytics are performed at HCOs, and only aggregated results are returned to the platform.

Available data include demographics, coded diagnoses (International Classification of Diseases (ICD), version 10), procedures (ICD-10 Procedure Coding System or Current Procedural Terminology), medications (National Library of Medicine and the Department of Veterans Affairs), and measurements (laboratory tests and body mass index (BMI), Logical Observation Identifiers Names and Codes).

Data Analysis

The analysis is based on:

a) *Query Builder*: Users select the relevant collaborative network from the Health Equity Research (HER) data. Information from registries and testing data is retrieved. Cohort construction involves specific inclusion and exclusion criteria, which are grouped according to international Coding Systems. Each criterion can be further refined using filters. Additional details can be integrated into each group, adding temporal constraints to define relationships between groups. Multiple groups can be used to define cohorts.

b) *Analytics*: Once the study cohort(s) have been constructed, by default, the analytics tool currently offers six distinct analysis options: "Analyse Outcomes", "Compare Outcomes", "Compare Cohorts", "Treatment Pathways", "Incidence and Prevalence", and "Advanced Explore Cohort". Baseline characteristics are analysed before matching. "Association" (risk ratio, risk difference, odds ratio (OR), "Kaplan–Meier Analysis" (log-rank test, hazard ratio (HR)), "Number of Instances", "Lab Results", and follow-up time for each cohort can also be retrieved.

Cohorts

<u>Cohort 1:</u>

Main cohort (C1) of people older than 40 years with a coded administrative diagnosis of COPD without any prior diagnosis of ARF or CRF.

Patients must have:

- Age (Age) (at least 40 years (most recent occurrence)).
- COPD: other chronic obstructive pulmonary *disease (UMLS:ICD10CM:J44), occurred at any time*.

Before the first instance of COPD, patients cannot have any of the following:

- Respiratory failure, unspecified (UMLS:ICD10CM:J96.9);
- Acute and chronic respiratory failure (UMLS:ICD10CM:J96.2);
- Acute respiratory failure (UMLS:ICD10CM:J96.0);
- Chronic respiratory failure (UMLS:ICD10CM:J96.1).

Cohort 2:
C2 cohort consisted of people older than 40 years with COPD who were subsequently diagnosed with CRF and without any prior diagnosis ARF.
Patients must have:
- Age (Age) (at least 40 years (most recent occurrence)).
- COPD other chronic obstructive pulmonary disease (UMLS:ICD10CM:J44), occurred at any time.
- CRF after COPD: chronic respiratory failure (UMLS:ICD10CM:J96.1), occurred at least 1 day after the first instance of COPD.

Before the first instance of COPD, patients cannot have any of the following:
- Acute respiratory failure (UMLS:ICD10CM:J96.0);
- Respiratory failure, unspecified (UMLS:ICD10CM:J96.9);
- Acute and chronic respiratory failure (UMLS:ICD10CM:J96.2).

Cohort 3:
C3 cohort consisted of people with COPD who were subsequently diagnosed with ARF and without any prior diagnosis CRF.
Patients must have:
- Age (Age) (at least 40 years (most recent occurrence)).
- COPD: other chronic obstructive pulmonary disease (UMLS:ICD10CM:J44), occurred at any time.
- ARF: acute respiratory failure (UMLS:ICD10CM:J96.0) or respiratory failure, unspecified (UMLS:ICD10CM:J96.9), occurred at least 1 day after the first instance of COPD.

Before the first instance of COPD, patients cannot have:
- Chronic respiratory failure (UMLS:ICD10CM:J96.1).

Covariates

Data of age, sex, coded comorbidities, respiratory support and ventilation procedures, BMI and medications were collected. No reliable data were available on the use of oxygen therapy or NIV in acute or home settings.

Diagnoses

1. Nicotine dependence (F17).
2. Essential (primary) hypertension (I10).
3. Chronic kidney disease (N18.5).
4. Ischemic heart diseases (I20 – I 25).
5. Acute myocardial infarction (I21).
6. Atrial fibrillation and flutter (I48).
7. Unspecified atrial flutter (I48.92).
8. Acute on chronic diastolic (congestive) heart failure (I50.33).
9. Neoplasms (C00-D49).

Drugs

10. Inhaler anti-inflammatories (RE101).
11. Inhaler sympathomimetic bronchodilators (RE102).
12. Oral sympathomimetic bronchodilators (RE103).
13. Oral xanthine bronchodilators (RE104).
14. Inhaler anticholinergic bronchodilators (RE105).
15. Oxygen (7806).

Procedures

16. Introduction of other therapeutic substance into respiratory tract via natural or artificial opening (3E0F7GC).
17. Assistance with respiratory ventilation, less than 24 consecutive hours, continuous positive airway pressure (5A09357).
18. Assistance with respiratory ventilation, 24-96 consecutive hours, continuous positive airway pressure (5A09457).

19. Assistance with respiratory ventilation, greater than 96 consecutive hours, continuous positive airway pressure (5A09557).
20. Assistance with respiratory ventilation, less than 24 consecutive hours, intermittent positive airway pressure (5A09358).
21. Assistance with respiratory ventilation, 24-96 consecutive hours, intermittent positive airway pressure (5A09458).
22. Assistance with respiratory ventilation, greater than 96 consecutive hours, intermittent positive airway pressure (5A09558).
23. Respiratory ventilation, 24-96 consecutive hours (5A1945Z).
24. Respiratory ventilation, greater than 96 consecutive hours (5A1955Z).
25. Respiratory ventilation, less than 24 consecutive hours (5A1935Z).

Analyse Outcome Function

The Analyse Outcomes Analytic supports four types of analyses: risk, survival, number of instances, and lab result distribution. The first three analyses support the "exclude patients with outcomes prior to the window" setting. This option can exclude patients from the analysis if they are not at risk for an outcome (e.g., if the outcome is a chronic disease). When "exclude patients with the outcome prior to the time window" is not checked, all patients in the cohort are included in the analysis, regardless of whether they had the outcome prior to the time window. When "exclude patients with the outcome prior to the time window" is checked, patients are excluded from the analysis if their record includes the outcome prior to the beginning of the time window. This selection will exclude all patients with the outcome prior to the index event. If the start of the time window for the analysis falls some days after the index event, patients will also be excluded if they have the outcome between the index event and the start of the time window.

<u>Risk Analysis</u>

The Risk Analysis calculates the fraction of patients with the selected outcome. The output summary includes: patients in the cohort (count of patients meeting query criteria); Patients with outcome in the cohort (of the patients in the cohort, count of patients that had the outcome in the time window); and risk (the fraction of patients in the cohort that have the outcome in the time window, i.e., patients with outcome/patients in cohort). The bar chart shows the risk of the outcome for the cohort.

Kaplan–Meier Analysis

The Kaplan–Meier Analysis estimates probability of the outcome at a respective time interval (daily time interval is used in this analysis). In order to account for the patients who exited the cohort during the analysis period and therefore should not be included in the analysis, censoring is applied. In this analysis, patients are removed from the analysis (censored) after the last fact in their record.

The output summary includes: patients in the cohort (count of patients meeting query criteria); patients with outcome (of the patients in the cohort, count of patients that had the outcome in the time window); median survival (the number of days when the survival drops below 50%; the "-" indicates that survival does not drop below 50% during the time window); and survival probability at the end-of-time window (the % survival at the end of the time window).

<u>Number of Instances Analysis</u>

The Number of Instances Analysis calculates how many times the outcome occurred in the time window. This analysis includes two additional settings: include patients with zero instances and the definition of an instance. Selecting to exclude patients with zero instances will remove these patients from the calculations for mean number of instances, standard deviation, or median. The histogram showing the distribution of patients by number of instances will not contain a bar for zero. Alternatively, by selecting to include patients with zero instances, the mean, standard deviation, and median for number of instances will reflect these patients. The histogram will contain a bar for zero patients.

The definition of an instance affects how counts are analysed. By selecting a date, each calendar date on which any of the terms selected in the outcome are recorded will represent one instance. For example, if the outcome is "Med A or Med B," and a patient has "Med A" on January 3, then both medications on January 4, then "Med B" on January 6, then that patient is considered to have three instances—January 3, January 4, and January 6.

Note that if an outcome occurs across several dates (e.g. visit: inpatient encounter), then only the start date is tracked for the purpose of counting instances. A patient who begins at stay on January 1, ends that stay on January 3, begins another stay on January 10, and ends that stay on January 15, is considered to have two instances of the outcome.

Selecting visit as an instance will count any visit that includes the outcome as one instance, regardless of how many times it occurred. For instance, consider a patient administered an analgesic on each of the three days that make up an inpatient stay following some index event. If analgesic is an outcome, these three administrations will represent only one instance, because all three are associated with the same visit.

The output summary includes: patients in cohort (count of patients meeting query criteria); patients with outcome (of the patients in the cohort, count of patients that had the outcome in the time window); mean (mean of the counts); standard deviation (standard deviation of the counts); median (median of the counts); and median (1+ instances) when patients with zero instances included in the analysis.

Laboratory Results Analysis

Lab results can be included in the analysis only for the outcomes that are labs. Only the most recent lab values in the time window are included. For the lab results that are numeric, the outcome summary includes: patients in cohort (count of patients meeting query criteria); patients with outcome (of the patients in the cohort, count of patients that had the outcome in the time window); mean (mean of the counts); and standard deviation (the standard deviation for lab values across patients in the cohort).

For the non-numeric lab results, three values are reported: counts of negative; positives; and unknowns.

The counts are represented in the bar chart as percentages of the total counts.

Characterisation Table

For large cohorts, there can be a long tabulation time to compile the data presented. In order to improve performance times, large cohort results are limited to approximately 10,000 patients per healthcare organisation (HCO). This sample is arbitrary; it is not a true random sample.

When partial results are presented:

- Users will see a note at the top of the page, stating that results are for a subset of the cohort;
- Patient counts in the results will reflect that subset.

In addition, results exclude patients who met the index event criteria more than twenty years ago. For most cohorts, a small number of patients are excluded; the vast majority of data in TriNetX comes from patient encounters that occurred in the last 20 years.

Of the 3,358,425 participants with COPD, 170,479 were excluded because they met the index event criteria more than 20 years ago. Cohort 1 therefore refers to 3,187,946 participants. Although TrinetX calculated demographic data for the full total, for characterisation Table 1, to simplify the analysis as explained above, only 935,483 participants were considered as a representative sample of Cohort 1.

Sensitivity Analysis

Sensitivity analysis is a methodological technique used to assess the extent to which changes in the input parameters of a model or analysis affect the results obtained. It is a fundamental tool for examining the robustness of a statistical or computational model with respect to uncertainties in data, assumptions, or methodological choices.

In this study, sensitivity analysis was used to verify the stability and consistency of the results obtained from analyses based on real-world data (RWD). Specifically, we computed the risk of ARF and CRF in the COPD cohort excluding outcome events occurring immediately after the index event to reduce the likelihood of including pre-existing conditions. Analyses were repeated on 1 day and 6 months after the index event to evaluate the temporal effect.

Results

Sensitivity analysis, conducted excluding participants who had developed the outcome in the first six months after COPD diagnosis, showed a reduction in the overall number of events and a reduction in the probability of outcome in all cohorts considered.

In the overall cohort of subjects with COPD, the risk of developing ARF was high in the early stages after diagnosis, with a probability of survival ranging from 54.754% (1-day window) to 57.11% (6-month window). A similar trend, although less marked, was observed for CRF, with an increase in survival probability from 78.144% to 79.094%.

Among COPD participants who subsequently developed CRF, the risk of ARF was particularly high in the months immediately following the diagnosis of CRF, with survival probability increasing from 13.224% to 23.391% when the observation window was shifted to six months.

Similarly, in COPD participants who had developed an episode of ARF, the probability of remaining free of CRF increased from 58.48% to 73.589% after excluding early events.

Overall, these results indicate that a substantial proportion of ARF episodes and CRF occur in the first few months after COPD diagnosis or the onset of the previous respiratory event, suggesting the existence of a period of particular clinical vulnerability in the early stages of the natural history of the disease.

Full results are reported **in Table 1SM.**

Table S1. Sensitivity Analysis

	Participants (n)		Participants presenting outcomes (n)		Probability of being free from the outcome (%)	
	at 1st day after the index event	at 6 months after the index event	at 1st day after the index event	at 6 months after the index event	at 1st day after the index event	at 6 months after the index event
**Patients with a recent COPD diagnosis**						
**ARF**	3,187,946	3,075,539	507,177	391,602	**54.754%**	57.11%
**CRF**	3,187,946	3,158,553	192,496	159,460	**78.144%**	79.094%
**Patients with COPD and a recent CRF diagnosis**						
**ARF**	232,581	90,352	118,741	25,019	**13.224%**	23.391%
**Patients with COPD and a recent ARF event**						
**CRF**	493,644	334,569	95,716	17,436	**58.48%**	73.589%
